# Identification of Neutrophil-Related Factor LCN2 for Predicting Severity of Patients With Influenza A Virus and SARS-CoV-2 Infection

**DOI:** 10.3389/fmicb.2022.854172

**Published:** 2022-04-12

**Authors:** Zhisheng Huang, Hui Li, Shuai Liu, Ju Jia, Ying Zheng, Bin Cao

**Affiliations:** ^1^Graduate School of Peking Union Medical College, Chinese Academy of Medical Sciences, Peking Union Medical College, Beijing, China; ^2^Department of Pulmonary and Critical Care Medicine, Center for Respiratory Diseases, China-Japan Friendship Hospital, Beijing, China; ^3^Department of Respiratory and Critical Care Medicine, Shandong Provincial Hospital Affiliated to Shandong First Medical University, Jinan, China; ^4^China-Japan Friendship Hospital, National Clinical Research Center for Respiratory Diseases, Clinical Center for Pulmonary Infections, Capital Medical University, Beijing, China

**Keywords:** severe infection, inflammatory response, Lipocalin 2, IAV, SARS-CoV-2

## Abstract

**Background:**

Influenza and COVID-19 are respiratory infectious diseases that are characterized by high contagiousness and high mutation and pose a serious threat to global health. After Influenza A virus (IAV) and SARS-CoV-2 infection, severe cases may develop into acute lung injury. Immune factors act as an important role during infection and inflammation. However, the molecular immune mechanisms still remain unclear. We aimed to explore immune-related host factors and core biomarker for severe infection, to provide a new therapeutic target of host factor in patients.

**Methods:**

Gene expression profiles were obtained from Gene Expression Omnibus and the Seurat R package was used for data process of single-cell transcriptome. Differentially expressed gene analysis and cell cluster were used to explore core host genes and source cells of genes. We performed Gene Ontology enrichment, Kyoto Encyclopedia of Genes and Genomes analysis, and gene set enrichment analysis to explore potential biological functions of genes. Gene set variation analysis was used to evaluate the important gene set variation score for different samples. We conduct Enzyme-linked immunosorbent assay (ELISA) to test plasma concentrations of Lipocalin 2 (LCN2).

**Results:**

Multiple virus-related, cytokine-related, and chemokine-related pathways involved in process of IAV infection and inflammatory response mainly derive from macrophages and neutrophils. LCN2 mainly in neutrophils was significantly upregulated after either IAV or SARS-CoV-2 infection and positively correlated with disease severity. The plasma LCN2 of influenza patients were elevated significantly compared with healthy controls by ELISA and positively correlated with disease severity of influenza patients. Further bioinformatics analysis revealed that LCN2 involved in functions of neutrophils, including neutrophil degranulation, neutrophil activation involved in immune response, and neutrophil extracellular trap formation.

**Conclusion:**

The neutrophil-related LCN2 could be a promising biomarker for predicting severity of patients with IAV and SARS-CoV-2 infection and may as a new treatment target in severe patients.

## Introduction

Respiratory virus infection represented by influenza and COVID-19 poses a serious threat to global health. Several influenza pandemics happened since the last century, including Spanish influenza in 1918, Asian influenza in 1957, Hong Kong influenza in 1968, and swine influenza in 2009, which have caused serious damage to the global economy and human health (Ziegler et al., 2018). SARS-Cov-2 (Li et al., 2021), a new coronavirus, first reported in 2020 by China, has been spreading around the world for nearly 2 years. By December 31, 2021, a total of 281,808,270 confirmed cases and 5,411,759 deaths have been reported globally by WHO. As the virus evolves, new strains emerge gradually, such as alpha, beta, delta, and omicron, which make the existing vaccine less effective (Callaway, 2021). Options for the treatment of both influenza and COVID-19 are currently limited. Moreover, the effect of antiviral therapy is affected by the timing of administration and emergence of drug-resistant strains (Lampejo, 2020).

Common respiratory viruses including Influenza A virus (IAV) and SARS-Cov-2 belong to RNA virus, and they have limited protein-coding capacity and rely on host factors for replication to complete the life cycle. Meanwhile, expression of host factors is altered by virus stimulation during the process of virus infection. Interaction between hosts and the virus determines prognosis of the disease (Ito et al., 2011; Mahler et al., 2021). Although an effective immune response is indispensible for eliminating virus, exaggerated response characterized by the so-called “cytokine storm” can trigger severe immune pathological damage (Karki et al., 2021; Rappe et al., 2021). Changes in the expression levels of host factors and abnormal immunity, such as cytokine storm (Liu et al., 2016; Guo and Thomas, 2017), lymphopenia (Cheng et al., 2019; Zhang et al., 2020b), and T cell exhaustion (Valero-Pacheco et al., 2013; Diao et al., 2020), during virus infection have been reported. Because of the conserved feature, host factors targeting therapy offers a new approach for the treatment of respiratory viral infection, and it is expected to limit viral replication, alleviate immune-mediated damage, and decrease mortality in severe infection hosts. In recent decades, series host factors have been found to have potential therapeutic value. However, the accurate key molecules and origin cells of host factors remain unclear.

Single-cell transcriptome sequencing (scRNA-seq) has recently emerged as a powerful method to identify cell types and analyze functional status at single-cell level (Dal Molin and Di Camillo, 2019). scRNA-seq is widely used in various fields including cancer (Suvà and Tirosh, 2019), developmental biology (Griffiths et al., 2018), and microbiology (Imdahl and Saliba, 2020) and has become a promising research tool in the fields of life sciences. scRNA-seq results of COVID-19 find that changes of host factors including EN-RAGE, TNFSF14, oncostatin M, ANXA1, FPR1, and S100A8/9 are correlated with disease severity, revealing the possible pathogenesis of severe COVID-19 and potential host therapeutic targets (Arunachalam et al., 2020; Guo et al., 2021; Ren et al., 2021).

To systematically identify the role of host factors in respiratory virus infection, we analyzed the public transcriptomics data of IAV and SARS-CoV-2 infection to further understand changes in host functions and identify important host factors in mice and human. We found that Lipocalin 2 (LCN2) increased in influenza and COVID-19 and positively correlated with disease severity of infected patients, which was associated with neutrophil degranulation, neutrophil associated inflammation, and neutrophil extracellular trap (NET) formation by bioinformatics functional analysis. The plasma LCN2 of clinical influenza patients was elevated significantly compared with healthy controls by enzyme-linked immunosorbent assay (ELISA) and positively correlated with disease severity of influenza patients. Therefore, neutrophil-related LCN2 might be an early biomarker for predicting severity of disease and provide a new method for therapy targeting of host factor in patients with severe IAV and SARS-CoV-2 infection.

## Materials and Methods

### Dataset Acquisition

The scRNA-seq data, microarray data, and RNA-seq data obtained from Gene Expression Omnibus (GEO)^1^ were included in this study for analysis. The selection criteria of mice datasets were as follows: (1) inclusion lung tissue or whole-blood samples from IAV infection and control mice within 3 days post infection (dpi), datasets involved with other factors (drug intervention and gene knockout) were excluded; (2) every group of dataset contained at least three reduplicate samples; and (3) with the mature of sequencing technology, datasets were selected in 2019–2021. On the basis of these criteria, GSE80011 and GSE124404 were obtained. However, GSE107947 was the only scRNA-seq data of influenza mice in GEO. Given that administration of aerosolized SARS-CoV-2 to K18-hACE2 mice could simulate the real situation well (Fumagalli et al., 2022), GSE184657 was the only dataset included in subsequent analysis of SARS-CoV-2. The scRNA-seq data (GSE107947) of four lung samples of mice were composed of one control sample, two influenza samples (A/PR/8) at 48 hours post infection (hpi), and one influenza sample at 72 hpi, containing 3,840 CD45-negative (CD45-) cells and 3,840 CD45-positive (CD45 +) cells. The microarray dataset (GSE80011) contained three influenza (A/WSN/33) and three control lung samples of mice at 24 hpi. The RNA-seq profiles (GSE124404) of six blood samples included three control mice and three mice with influenza (A/PR/8) at 48 hpi. The RNA-seq profiles (GSE184657) contained four mice with SARS-Cov-2 infection and two control mice at 6 dpi.

The datasets of patients met the following criteria: (1) lung tissue or whole-blood samples from IAV and SARS-Cov-2 infection; (2) datasets contained at least 30 samples; and (3) patients contained various severity classifications. The clinical whole-blood samples of patients with influenza and COVID-19 were composed of GSE111368 and GSE157103. The severity of patients with influenza was divided into mild, moderate, and severe on the basis of whether supplemental oxygen and invasive mechanical ventilation was required. We acquired 130 healthy control samples, the earliest acute stage samples of 94 patients with pH1N1 infection and 44 paired convalescent samples for analysis from GSE111368. The COVID-19 dataset (GSE157103) contained 100 patients, which were recorded clinical information, such as need for admission into the intensive care unit (ICU) and requiring mechanical ventilation support, sequential organ failure assessment (SOFA) scores, and acute physiology and chronic health evaluation (APACHE II) score.

### Single-Cell RNA-seq Data Processing

The Seurat R package 4.0.0 was used for data filtering, statistical analysis, and exploration of the scRNA-seq data. We used the same rigorous method to process data of CD45- cells and CD45 + cells separately. First, we discarded low-quality cells on the basis of the quality control standards that every cell expressed > 200 genes and genes expressed in > 3 cells. Then, the gene expression of remaining cells was normalized, and 2,000 highly variable genes from each sample were identified by the vst method. All genes were scaled, and the principal component analysis was conducted. The cells were clustered by unsupervised clustering (resolution = 0.5) and visualized by t-distributed stochastic neighbor embedding (tSNE) using the top 10 principal components. Last, we annotated cell types through known marker gene in each cluster of CD45- cells and CD45 + cells. Differentially expressed genes (DEGs) were found using the FindMarkers in Seurat R package.

### Differentially Expressed Genes, Functional Enrichment Analysis, Gene Set Enrichment Analysis, and Gene Set Variation Analysis

To estimate the difference among different groups, DEGs were identified using the limma package in R software (version 4.0.4) and selected with the commonly used thresholds of fold change (FC) > 2 and adj. *p* value < 0.05. Gene Ontology (GO) and Kyoto Encyclopedia of Genes and Genomes (KEGG) pathway enrichment analysis were performed using clusterprofiler package in R software and *p* < 0.05 was considered statistically significant. We performed gene set enrichment analysis (GSEA) between patients with high LCN2 and low LCN2 with the R package clusterprofiler. To investigate intensity of response to virus and inflammatory response pathways in different cell types, we performed gene set variation analysis (GSVA) with GSVA R package.

### Patients and Clinical Information

The clinical study of patients with IAV infection included 25 healthy controls and 43 influenza patients from the Department of Pulmonary and Critical Care Medicine of China-Japan Friendship Hospital during influenza season of 2017–2021. The study was approved by China-Japan Friendship Hospital Ethics Committee (approval no. 2018-120-K86). The patients must meet the following criteria: (i) reverse transcription PCR testing of an appropriate respiratory tract sample was positive; (ii) the time from onset of influenza-like symptoms was ≤14 days; (iii) acute blood samples within 48 h after hospitalization were able to obtain; and (iv) age was > 18 years old. In accordance with the upper classification criteria of severity, severe influenza was defined with respiratory failure that needed invasive mechanical ventilation. Moderate influenza was defined with damage of respiratory function that required supplemental oxygen therapy, including nasal high-flow oxygen therapy and/or non-invasive mechanical ventilatory support. Mild influenza was defined without supplemental oxygen. Forty-three influenza patients contained eight mild patients, 19 moderate patients, and 16 severe patients. Important clinical information including age, sex, and incidence of acute respiratory distress syndrome (ARDS) were obtained from medical records.

### Enzyme-Linked Immunosorbent Assay

Lipocalin 2 expression levels of plasma were measured using the human LCN2 enzyme immunoassay kit (Elabscience, Wuhan, China) according to the manufacturer’s instructions.

### Statistical Analysis

The Student’s *t*-test or Mann–Whitney U test (*t*-test or wilcox test in R 4.0.4) was used to compare two group differences, as appropriate. *P < 0.05, ^**^P < 0.01, ^***^P < 0.001, and ^****^P < 0.0001 represent significant statistical differences.

## Results

### Inflammatory Response Mainly Derives From Macrophages and Neutrophils After IAV Infection

**FIGURE 1:**
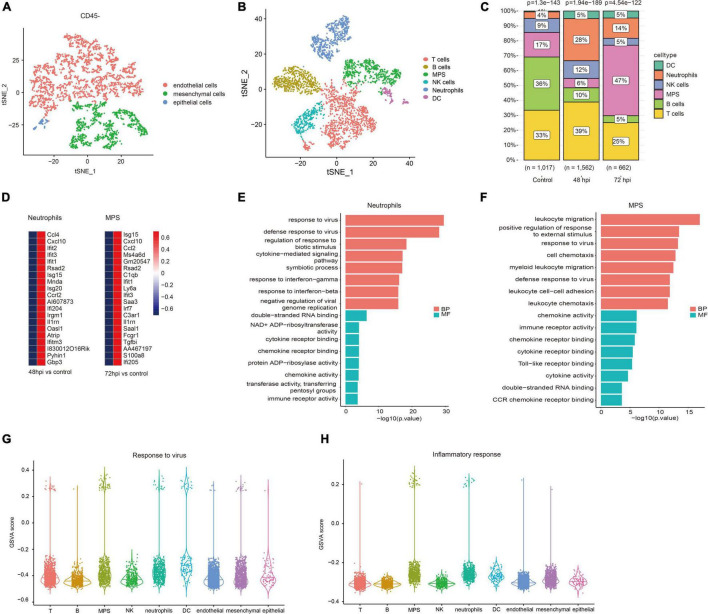
Immune landscape associated with IAV infection. Cellular subsets from CD45- **(A)** and CD45 + **(B)** cells in the lung (GSE107947). **(C)** Proportions of immune cells clusters in different studied subjects. **(D)** Top 20 upregulated genes of neutrophils at 48 hpi and MPS at 72 hpi. **(E)** Top eight GO terms of BP and MF in neutrophils. **(F)** Top eight GO terms of BP and MF in MPS. GSVA scores of response to virus **(G)** and inflammatory response **(H)** in different cell types.

According to quality control standards, 1,228 cells were excluded, and 6,452 cells were included in the scRNA-seq data GSE107947. These cells consisted of 3,211 CD45- cells and 3,241 CD45 + cells using flow cytometry sorting technology. To examine the host immune response characteristics in a cell type–specific manner, 3,211 CD45- cells and 3,241 CD45 + cells were subjected to tSNE on the basis of highly variable genes using the Seurat package. These cells were successfully classified into 10 different clusters unbiased separately (Supplementary Figure 1). These clusters of CD45- cells were assigned to three different cell types (Figure 1A), and these clusters of CD45 + cells were annotated to six different cell types (Figure 1B) on the basis of well-known marker genes (Supplementary Figure 2). The CD45- cells included endothelial cells, mesenchymal cells, and epithelial cells. CD45 + cells contained T cells, B cells, mononuclear phagocyte system (MPS), natural killer (NK) cells, neutrophils, and dendritic cells (DC). To illustrate immunological changes after influenza infection, we analyzed the dynamic change of relative proportions of immune cells in the lung tissue of mice (Figure 1C). After influenza infection, the percentage of MPS, neutrophils, and DC significantly increased, whereas that of B cells significantly decreased. In addition, the percentage of NK cells and T cells at 72 hpi decreased slightly compared with that at 48 hpi.

Neutrophils and macrophages were important immune cells that were involved in the important human host mechanism against microorganisms. In this study, neutrophils significantly increased at 48 hpi, and macrophages rose noticeably at 72 hpi. Interferon-stimulated genes (Ifit1, Rsad2, and Isg15), and chemokine (Cxcl10, Ccl2, and Ccl4) were significantly upregulated in neutrophils and macrophages (Figure 1D). GO enrichment analysis showed that DEGs were related to antiviral immune-related biological processes and cytokine/chemokine related molecular function in neutrophils (Figure 1E). The function analysis of macrophages was also related to leukocyte migration, antiviral immune, and cytokine/chemokine activity (Figure 1F). It is well known that a large number of cytokines (e.g., IL-1β, IL-6, and TNF-α) are produced in the process of inflammatory response and cytokine storm was correlated with disease severity and worse prognosis (Tisoncik et al., 2012). However, what kind of cells is mainly involved in the inflammatory response remains unclear. We evaluated GSVA score of response to virus and inflammatory response among all cell types. The results revealed that MPS and neutrophils showed a higher expression level in both response to virus and inflammatory response pathways (Figures 1G,H). It suggests that these cells may mediate hyper-inflammatory features and are an important source of cytokine storm after influenza infection.

### Expression of Lipocalin 2 in Neutrophils Was Upregulated After IAV Infection in Mice

**FIGURE 2:**
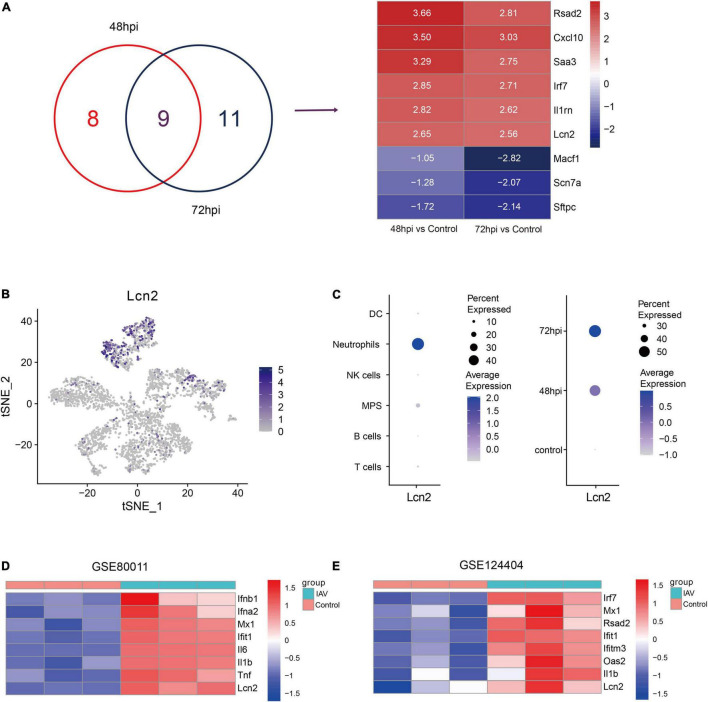
Identification and validation of key genes in IAV infection in public dataset. **(A)** Venn diagram to indicate 9 shared genes from DEGs and log2 fold change of shared genes (GSE107947). **(B)** Expression level of LCN2 in immune cells. **(C)** LCN2 expression level of neutrophils in different groups. **(D,E)** Validation of LCN2 in IAV infection.

To screen key genes in influenza, difference analysis was performed between control mice and IAV infection mice at 48 and 72 hpi. Top 10 upregulated and downregulated genes at 48 and 72 hpi were selected to take the intersection, and six overlapping upregulated genes were filtered, including Rsad2, Cxcl10, Saa3, Irf7, Il1rn, and LCN2 (Figure 2A). Rsad2, Cxcl10 (Yang et al., 2020), Saa3 (Ather et al., 2018), Irf7 (Hatesuer et al., 2017), and Il1rn (Schmitz et al., 2005), which have been reported in influenza, whereas the role of LCN2 in influenza was still unknown. We explored the distribution of LCN2 among all cell types using tSNE and found LCN2 mainly expressed in neutrophils (Figure 2B) and partially expressed in CD45- cells (Supplementary Figure 3). Moreover, expression of LCN2 in neutrophils was upregulated after influenza infection (Figure 2C), and it was also upregulated in little other myeloid cells (Supplementary Figure 4).

To verify this finding, the expression levels of LCN2 in mice infected with influenza virus were assessed with another two datasets (GSE80011 and GSE124404). The microarray dataset GSE80011 contained three influenza and three control lung tissues of mice at 24 hpi. As shown in Figure 2D, the expression levels of LCN2 was significantly higher in the lung tissues of mice infected with influenza virus, compared with that of the controls (Log_2_FC = 3.06, adj. *p* value = 0.0005). Meanwhile, the expression level of LCN2 in peripheral blood samples of mice infected with influenza virus was also upregulated (Log_2_FC = 2.75, adj. *p* value = 0.0003) (Figure 2E).

### Lipocalin 2 Expression Level in Peripheral Blood Positively Correlated With Disease Severity of Influenza Patients

**FIGURE 3:**
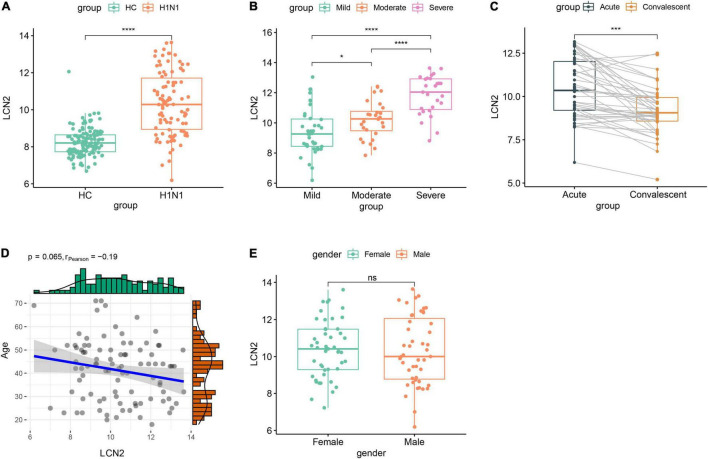
Correlation between LCN2 and clinical features of IAV infection in public dataset. **(A)** Expression level of LCN2 in healthy controls and patients with IAV infection (GSE111368). **(B)** Expression level of LCN2 in patients with mild, moderate, and severe influenza. **(C)** Expression level of LCN2 in acute and convalescent patients with IAV infection. **(D)** Association of patients’ age with expression level of LCN2. **(E)** Expression level of LCN2 in female and male patients with IAV infection.

We further investigated expression characteristics of LCN2 in patients. Ninety-four acute stage samples and 44 paired convalescent samples in GSE111368 dataset were involved in the analysis. The acute stage samples collected from 39 mild patients, 28 moderate patients, and 27 severe patients according to whether need supplemental oxygen and invasive mechanical ventilation. Compared with healthy controls, influenza-infected patients showed significantly higher level of LCN2 in peripheral blood leukocytes (Figure 3A). Furthermore, the expression level of LCN2 positively correlated with disease severity (Figure 3B). Paired samples revealed that the expression level of LCN2 declined at convalescent stage (Figure 3C). Expression of LCN2 was not influenced by age (Figure 3D) and gender (Figure 3E).

**FIGURE 4:**
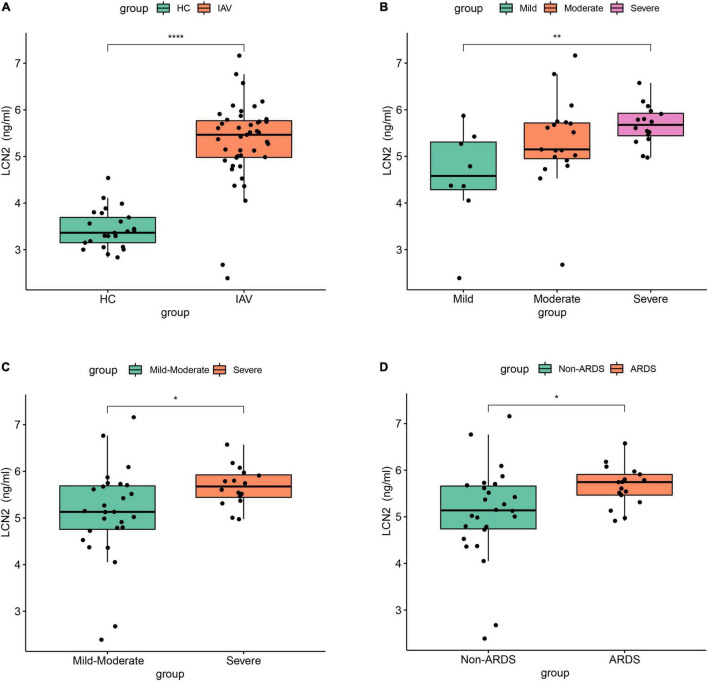
The association between LCN2 and disease severity in clinical patients with IAV infection. **(A)** Serum LCN2 levels in healthy controls and patients with IAV infection. **(B,C)** Serum LCN2 levels in patients with mild, moderate, and severe influenza. **(D)** Serum LCN2 levels in ARDS and non-ARDS patients with IAV infection.

To further clarify the expression level of LCN2 and its correlation with disease severity, we enrolled 43 patients with influenza infection, including eight mild patients, 19 moderate patients, and 16 severe patients. The protein level of LCN2 in plasma samples in acute phase was detected using ELISA. We found that the concentration levels of LCN2 in influenza patients were significantly higher than that in healthy controls (Figure 4A). Consistent with the above analysis, patients with severe illness had significantly higher LCN2 levels than those with mild or mild-moderate disease (Figures 4B,C). Patients with ARDS exhibited higher levels of LCN2 than those without (Figure 4D). Collectively, these findings suggested that plasma levels of LCN2 were positively correlated with severity of IAV infection and may be a clinically relevant biomarker for predicting severity of patients with IAV infection.

### Lipocalin 2 Was Involved in Functions of Neutrophils in Influenza Patients

**FIGURE 5:**
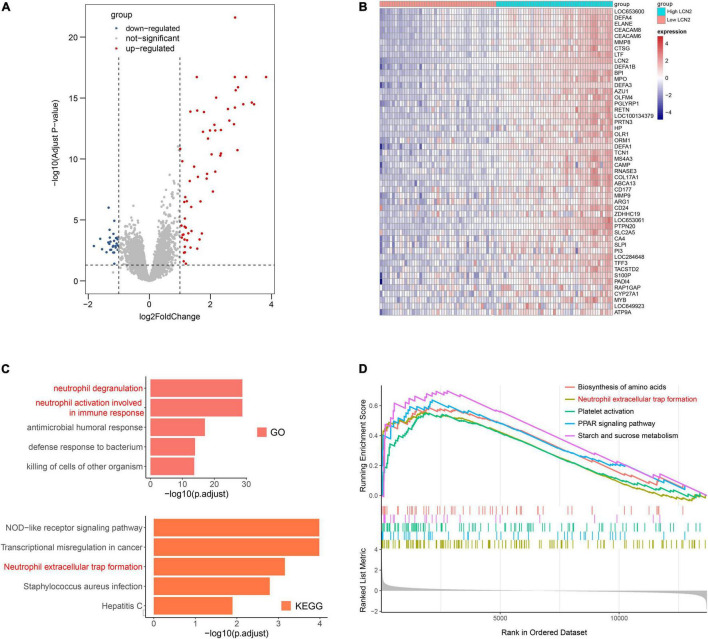
Functional analysis of LCN2 in patients with IAV. **(A)** DEGs between patients with low LCN2 and high LCN2 with IAV infection (GSE111368). **(B)** Heatmap of DEGs between patients with low LCN2 and high LCN2 with IAV infection. **(C)** GO terms and KEGG pathway enrichment analysis of DEGs between patients with low LCN2 and high LCN2 with IAV infection. **(D)** GSEA in groups with patients with high LCN2.

Because LCN2 was upregulated after influenza infection and correlated with disease severity, we further explored the possible function of LCN2. The influenza patients were equally divided into high-LCN2 group and low-LCN2 group on the basis of the median expression level of LCN2 in GSE111368. A total of 91 DEGs from the influenza patients with high LCN2 were identified by comparing with low LCN2. This total included 62 upregulated genes and 29 downregulated genes, which were indicated in the volcano plot shown in Figure 5A. The top 50 upregulated genes of DEGs were represented as a heatmap (Figure 5B). GO and KEEG pathway enrichment analysis of DEGs in LCN2-high samples identified an enrichment of genes in pathways related to neutrophils inflammation (neutrophil degranulation and neutrophil activation involved in immune response and antimicrobial humoral response) and NET formation (Figure 5C). Using GSEA analysis, we also found that LCN2 was associated with NET formation in influenza (Figure 5D).

### Similar Expression Pattern of Lipocalin 2 Was Found in Patients With COVID-19 and Mice

**FIGURE 6:**
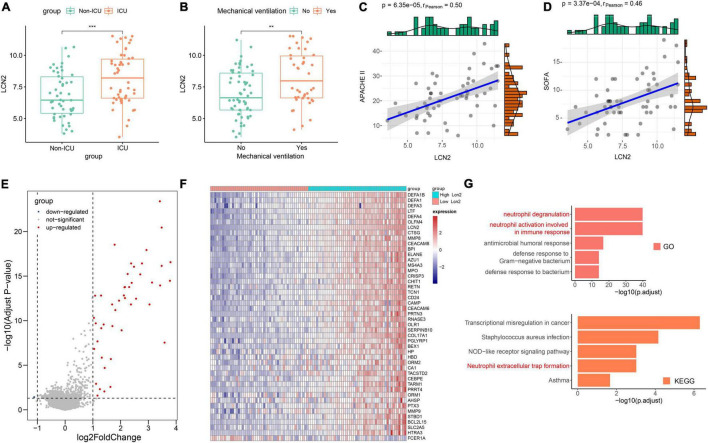
The role of LCN2 in patients with COVID-19. **(A)** Expression level of LCN2 in ICU and non-ICU patients with COVID-19 (GSE157103). **(B)** Expression level of LCN2 in patients with COVID-19 with mechanical ventilation and without mechanical ventilation support. **(C,D)** Correlation analysis between LCN2 and SOFA, APACHE II in patients with COVID-19. **(E)** DEGs between patients with low LCN2 and high LCN2 with COVID-19. **(F)** Heatmap of DEGs between patients with low LCN2 and high LCN2 with COVID-19. **(G)** GO terms and KEGG pathway enrichment analysis of DEGs between patients with low LCN2 and high LCN2 with COVID-19.

COVID-19 has some similarities in the course of disease development as influenza. The dataset GSE184657 showed the expression level of LCN2 in the lung tissues of mice infected with SARS-CoV-2 was significantly higher (Log_2_FC = 2.93, *p* = 0.0414) than that of the control at 6dpi (Supplementary Figure 5). Compared with patients in general wards, patients with COVID-19 at ICU showed higher level of LCN2 (Figure 6A). Similarly, the expression level of LCN2 was higher in patients who needed mechanical ventilation than those without (Figure 6B). APACHE II and SOFA scores, used to stratify the severity of illness, showed a positive correlation with the expression level of LCN2 in in leukocytes from whole blood (Figures 6C,D). Similarly, we found that LCN2 was involved in functions of neutrophils in patients with COVID-19, including neutrophil degranulation, neutrophil activation involved in immune response, and NET formation (Figures 6E–G).

## Discussion

Increasing evidence indicates that excessive immune response leads to immune-mediated damage and affects disease process and prognosis (Guo et al., 2021; Karki et al., 2021; Rappe et al., 2021). This emphasizes the importance of host factors in predicting clinical progression and prognosis in patients with viral infectious diseases. In this study, we found that a neutrophil-associated host factor LCN2 was significantly elevated and associated with severity in both influenza and patients with COVID-19 by integrated public data analysis. The increase of serum LCN2 level in clinical influenza patients was verified by ELISA and positively correlated with disease severity of influenza patients. Further bioinformatics functional analysis showed LCN2 might influence disease development by affecting neutrophil degranulation, neutrophil activation, and NET formation.

In acute respiratory virus–infected patients, hyper-inflammatory responses involving the release of pro-inflammatory cytokines (e.g., IL-1, IL-6, IL-8, and TNF) impair the function of lung and lead to severe complications (Andreakos et al., 2021). However, it is unclear which types of cells are responsible for the release of large number of cytokines in critical patients. Previous research has shown that inflammation-related factors (IL-1, TNF, CXCL1, CXCL2, S100A8, and S100A9) predominantly express in neutrophils, and neutrophils infiltration largely account for lethal influenza infection (Brandes et al., 2013). In this study, we found that immune response pattern transformed from early antiviral response into inflammatory response gradually with the elimination of influenza virus in the lung tissue. In addition, the inflammatory response was mainly mediated by neutrophils and mononuclear macrophages. These results are consistent with previous report (Zhang et al., 2020a) and suggest that neutrophils may play an important role in the development of severe disease in patients infected with IAV.

Lipocalin 2, also known as neutrophil gelatinase-associated lipocalin, or 24p3, is a secretory glycoprotein involved in various inflammatory processes (Kjeldsen et al., 2000), which is found in various cells, such as neutrophils, macrophages, epithelial cells, and adipocytes. Given that LCN2 is readily detected in plasma, urine, central nervous system, and various body fluids, it has been found to be associated with a variety of diseases (Moschen et al., 2017). In patients with adult-onset Still’s disease (AOSD), neutrophil-derived LCN2 is higher than healthy control in plasma and liver tissue, and it could serve as a potent biomarker for identifying liver damage caused by hyper-inflammatory state of AOSD (Jia et al., 2021). LCN2 is also upregulated in serum and liver tissue of patients with non-alcoholic fatty liver (NAFL), and serum LCN2 levels could be a novel biomarker for the diagnosis of non-alcoholic steatohepatitis (NASH), which may be participate in the transition from NAFL to NASH by mediating inflammation. Besides, LCN2 has been demonstrated as a biomarker for inflammatory bowel disease (Thorsvik et al., 2017), vascular dementia (Llorens et al., 2020), neuropsychiatric lupus (Mike et al., 2019), and subarachnoid hemorrhage outcome (Yu et al., 2021). The level of LCN2 could predict disease severity and survival in acute kidney injury (Bennett et al., 2008; Kümpers et al., 2010).

Research studies have revealed that LCN2 is also involved in infectious diseases. LCN2 attenuates bacterial growth by binding and sequestering the iron-scavenging siderophores to prevent bacterial iron acquisition (Goetz et al., 2002). LCN2 from both neutrophils and local epithelium can combat Klebsiella pneumoniae lung infection in mice (Cramer et al., 2017). Genetic deletion of LCN2 gene in neutrophils reduces the bactericidal effect of NETs (Li et al., 2018). However, Warszawska et al. (2013) report that LCN2 deactivates macrophages through an IL-10/STAT3-dependent mechanism and worsens pneumococcal pneumonia outcomes, which may be due to differences cell subset and bacterial strain. Besides, LCN2 can be as a biomarker of systemic inflammation in infants hospitalized with respiratory syncytial virus (RSV) infection (Sawatzky et al., 2019) and as a biomarker of inflammation, monocyte activation, and cardiac stretch during activation of the renin-angiotensin-aldosterone system in human immunodeficiency virus (Bogorodskaya et al., 2019). In this study, we found that neutrophil is one of the main drivers of inflammatory response after virus infection. As an important functional gene of neutrophil, LCN2 was upregulated in both plasma and lung tissue after IAV and SARS-CoV-2 infection. Plasma LCN2 level was positively associated with disease severity. Therefore, neutrophil-related LCN2 might serve as a potential biomarker for predicting disease severity in both patients with influenza and COVID-19. Meanwhile, we also found that the difference between mild and severe clinical patients had not multiplied obviously compared with the difference in mice. This difference may be caused by time point of clinical infection patients, and patients come to the hospital only when the apparent symptoms appear, as neutrophils are generated enriched at early infection and LCN2 is mainly expressed in neutrophils.

Lipocalin 2 is involved in various biological processes, including immune inflammatory response and iron homeostasis (Xiao et al., 2017). LCN2 acts as a scavenger of bacterial siderophores and has antibacterial activity against siderophores dependent bacteria (Saiga et al., 2008). In addition, LCN2 can also interact with LCN2 receptor 24p3R of neutrophils to activate ERK pathway and regulate neutrophil migration and adhesion (Schroll et al., 2012). In the study, functional analysis demonstrated that LCN2 was associated with NET formation, which may reveal that LCN2 influences the development of disease through NETs. NETs, composed of a scaffold of DNA with histones and cytotoxic neutrophil-derived proteins, are released by neutrophils to contain invading microbes during infection and inflammation (Brinkmann et al., 2004). NETs were originally thought to eliminate bacteria but, now, are proved to protect against many viral pathogens, including influenza virus, RSV, and dengue virus (Schönrich and Raftery, 2016). NETs capture viral particles through the NETs structure and eliminate the virus through high concentration MPO and defensin or prevent the spread of the virus (Brinkmann and Zychlinsky, 2007). Therefore, the appropriate level of LCN2 possesses a protective role in mild virus infection (Watzenboeck et al., 2021). Nevertheless, NETs not only can eliminate invading pathogens but can also harm the host as well. The high level of NETs is able to kill epithelial and endothelial cells (Saffarzadeh et al., 2012). The level of NETs is higher in severe than that in mild influenza patients and is significantly associated with APACHE II score, multiple organ dysfunction syndrome (MODS), and patient survival rate of clinical influenza patients (Zhu et al., 2018). The high level of NETs is also closely associated with thrombosis in patients with COVID-19 (Middleton et al., 2020). At present, the role and exact molecular mechanism of LCN2 are still unclear and need to be further explored in severe viral infection.

There are limitations in this study. In the validation of clinical samples, we only detected the expression level of LCN2 in the plasma of patients, whereas the expression level of LCN2 in the alveolar lavage fluid of patients is unclear. In recent years, because there are fewer patients with influenza and COVID-19 in China, the sample size is small. In future studies, we will try to collect bronchoalveolar lavage fluid (BALF) from patients as much as possible and increase the sample size of the study to improve the stability of the results.

## Conclusion

In conclusion, we identify that a host factor LCN2 associated with neutrophils through comprehensive analysis of public database and data from clinical influenza patient samples could be used as potential biomarkers of predicting severity of patients with IAV and SARS-CoV-2 infection and provide a new direction for targeted treatment of host factor in respiratory virus infection. However, the specific mechanism of LCN2 is still unclear, which needs to be further verified by molecular biology experiments.

## Data Availability Statement

Publicly available datasets used in the study were downloaded from GEO database: GSE107947, GSE80011, GSE124404, GSE184657, GSE111368, and GSE157103.

## Ethics Statement

The studies involving human participants were reviewed and approved by China-Japan Friendship Hospital Ethics Committee. The patients/participants provided their written informed consent to participate in this study.

## Author Contributions

BC: conception and design. ZH, SL, JJ, and YZ: acquisition of data. ZH and HL: analysis and interpretation of data. ZH: writing the draft. BC, HL, and SL: revising of manuscript. All authors contributed to the article and approved the submitted version.

## Conflict of Interest

The authors declare that the research was conducted in the absence of any commercial or financial relationships that could be construed as a potential conflict of interest.

## Publisher’s Note

All claims expressed in this article are solely those of the authors and do not necessarily represent those of their affiliated organizations, or those of the publisher, the editors and the reviewers. Any product that may be evaluated in this article, or claim that may be made by its manufacturer, is not guaranteed or endorsed by the publisher.

## Footnotes

1
http://www.ncbi.nlm.nih.gov/geo/
