# Supplementary figures and images for: Identification of Neutrophil-Related Factor LCN2 for Predicting Severity of Patients With Influenza A Virus and SARS-CoV-2 Infection
Source: Front Microbiol. 2022 Apr 12;13:854172. doi: 10.3389/fmicb.2022.854172 (PMC9039618; doi:10.3389/fmicb.2022.854172)

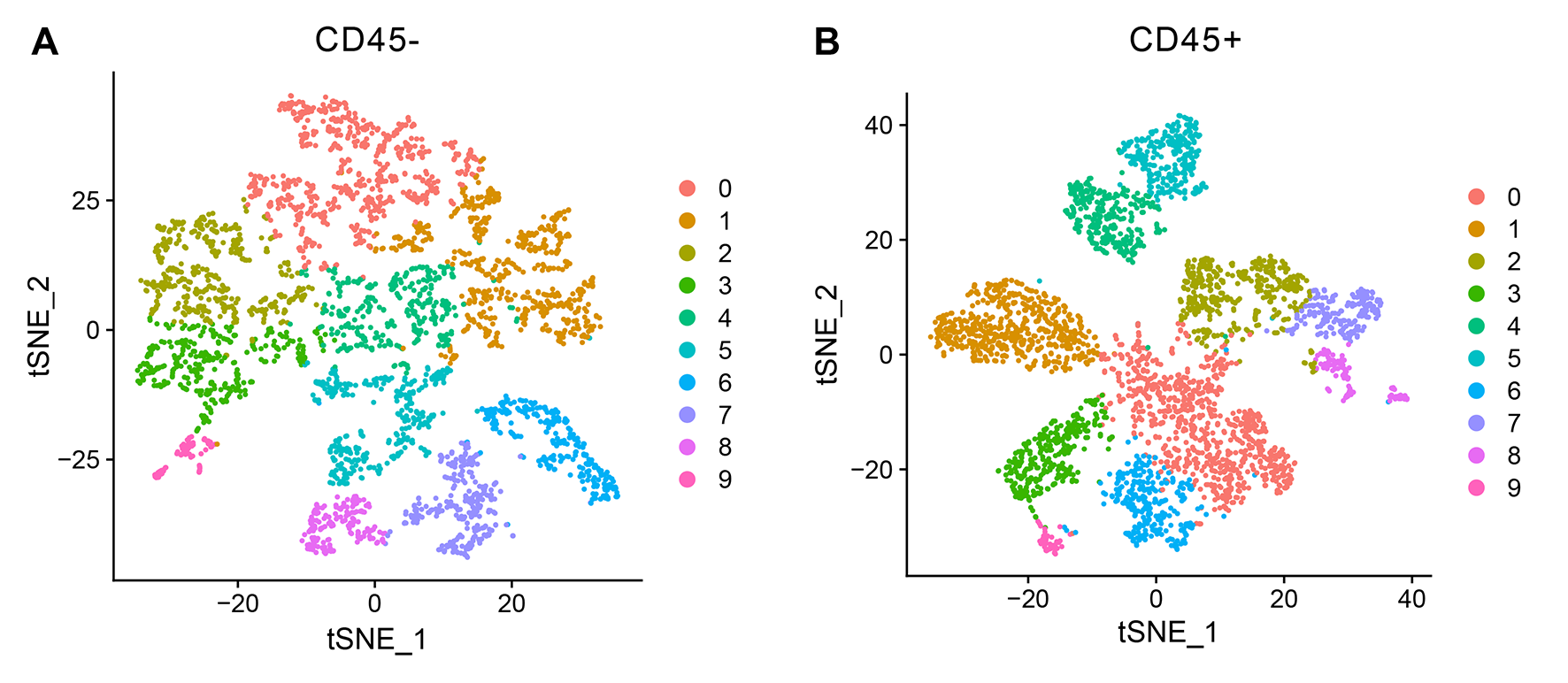

Supplement: Supplementary file 1 [file Image_1.TIF]

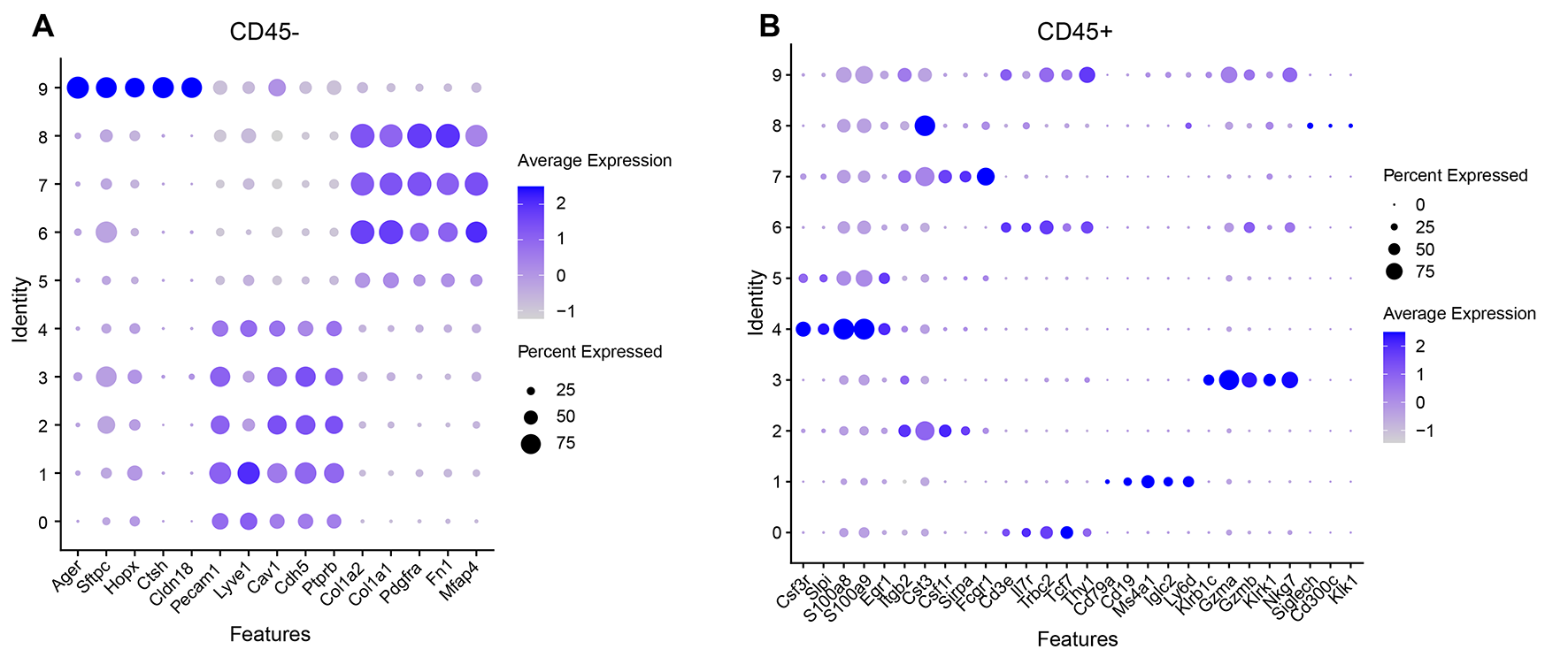

Supplement: Supplementary file 2 [file Image_2.TIF]

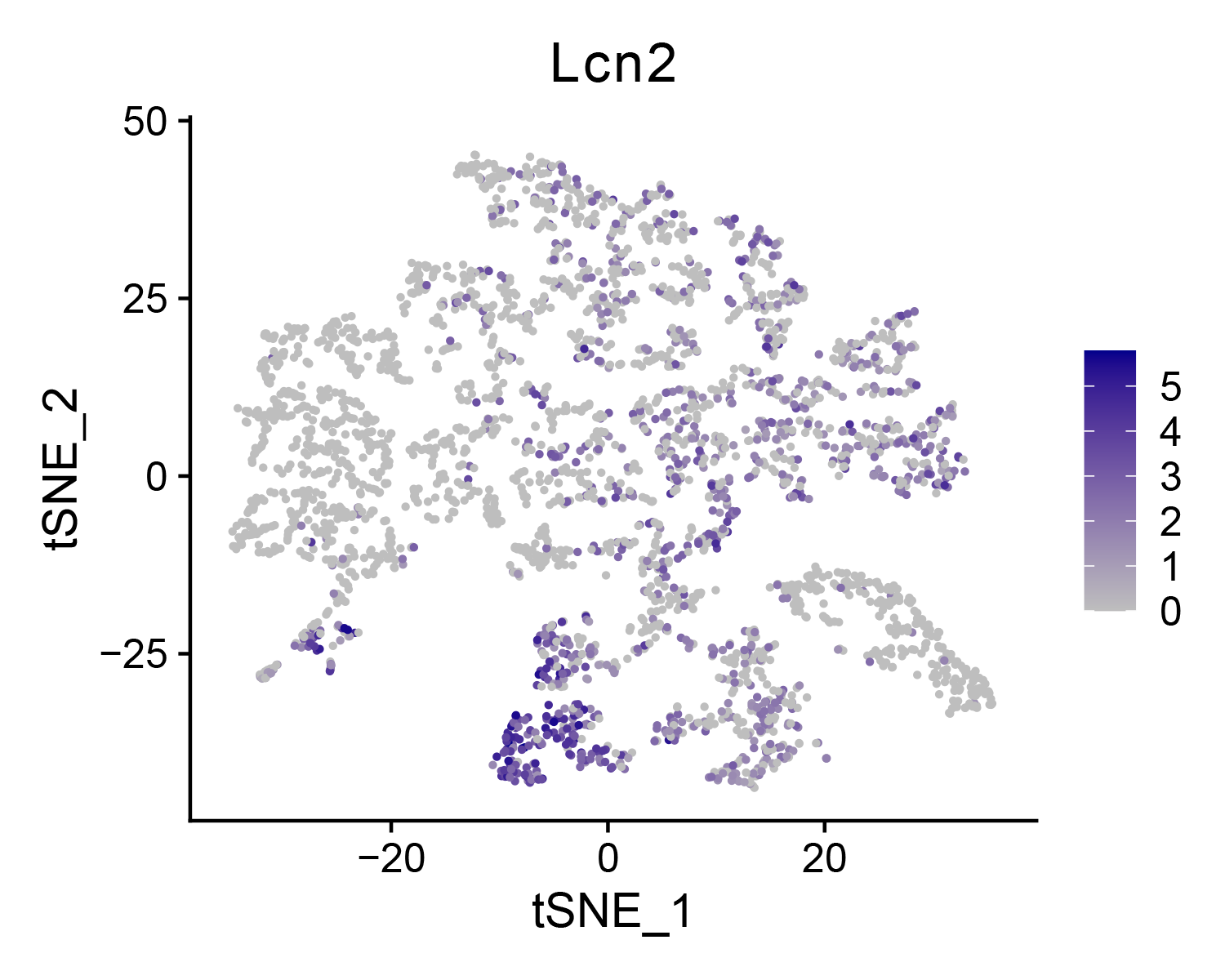

Supplement: Supplementary file 3 [file Image_3.TIF]

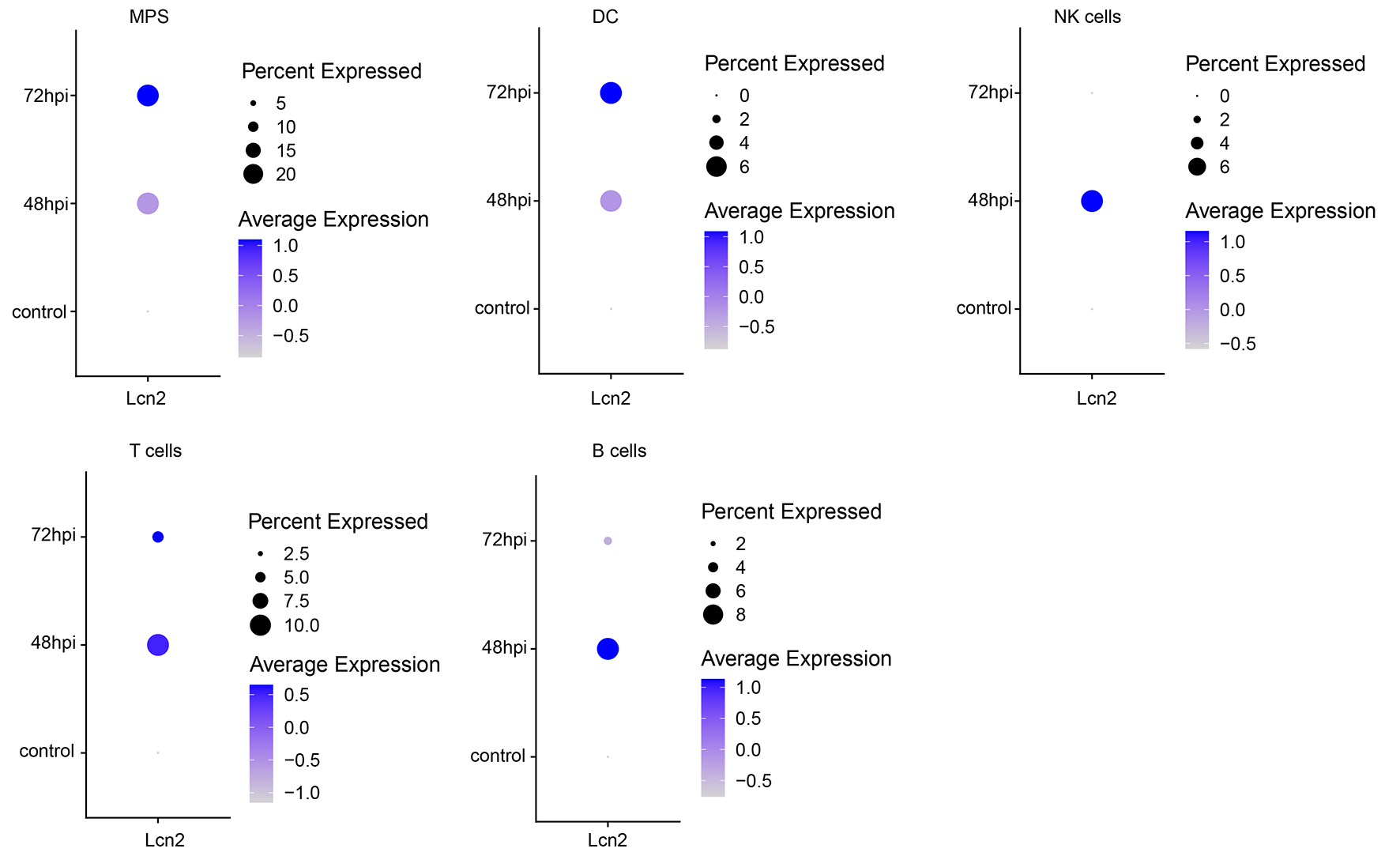

Supplement: Supplementary file 4 [file Image_4.TIF]

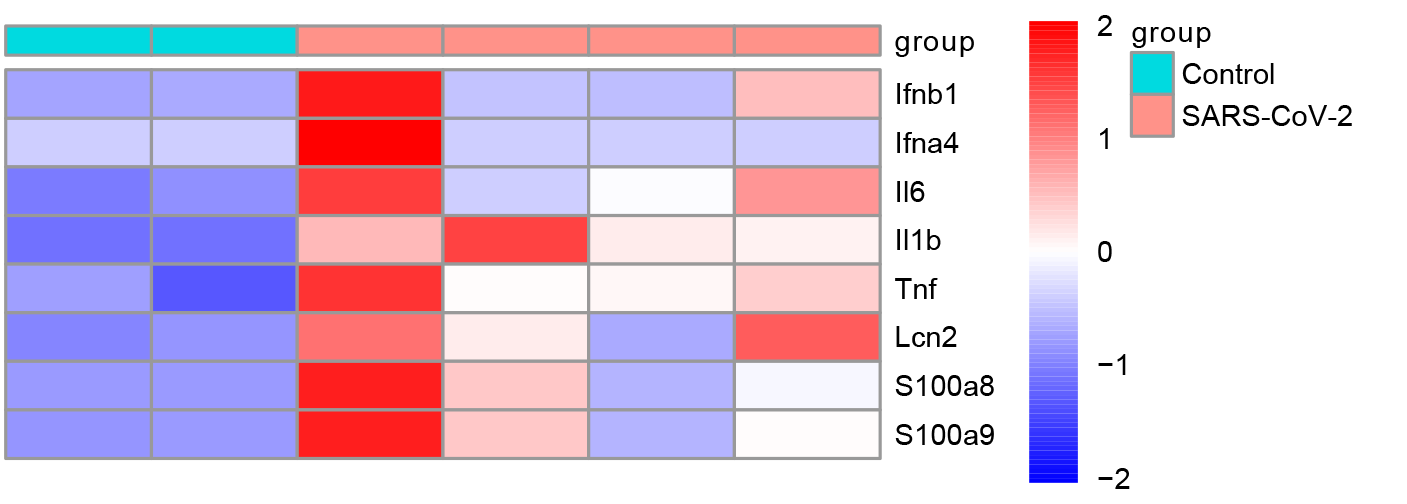

Supplement: Supplementary file 5 [file Image_5.TIF]
